# Facial mimicry and the mirror neuron system: simultaneous acquisition of facial electromyography and functional magnetic resonance imaging

**DOI:** 10.3389/fnhum.2012.00214

**Published:** 2012-07-26

**Authors:** Katja U. Likowski, Andreas Mühlberger, Antje B. M. Gerdes, Matthias J. Wieser, Paul Pauli, Peter Weyers

**Affiliations:** Department of Psychology, University of WürzburgGermany

**Keywords:** mimicry, EMG, fMRI, mirror neuron system

## Abstract

Numerous studies have shown that humans automatically react with congruent facial reactions, i.e., facial mimicry, when seeing a vis-á-vis' facial expressions. The current experiment is the first investigating the neuronal structures responsible for differences in the occurrence of such facial mimicry reactions by simultaneously measuring BOLD and facial EMG in an MRI scanner. Therefore, 20 female students viewed emotional facial expressions (happy, sad, and angry) of male and female avatar characters. During picture presentation, the BOLD signal as well as *M. zygomaticus major* and *M. corrugator supercilii* activity were recorded simultaneously. Results show prototypical patterns of facial mimicry after correction for MR-related artifacts: enhanced *M. zygomaticus major* activity in response to happy and enhanced *M. corrugator supercilii* activity in response to sad and angry expressions. Regression analyses show that these congruent facial reactions correlate significantly with activations in the IFG, SMA, and cerebellum. Stronger zygomaticus reactions to happy faces were further associated to increased activities in the caudate, MTG, and PCC. Corrugator reactions to angry expressions were further correlated with the hippocampus, insula, and STS. Results are discussed in relation to core and extended models of the mirror neuron system (MNS).

## Introduction

Humans tend to react with congruent facial expressions when looking at an emotional face (Dimberg, 1982). They react, for example, with enhanced activity of the *M. zygomaticus major* (the muscle responsible for smiling) when seeing a happy expression of a vis-á-vis' person or with an increase in *M. corrugator supercilii* (the muscle involved in frowning) activity in response to a sad face. Such facial mimicry reactions occur spontaneously and rapidly already after 300–400 ms (Dimberg and Thunberg, 1998) and even in minimal social contexts (Dimberg, 1982; Likowski et al., 2008). They appear to be automatic and unconscious, because they occur without awareness or conscious control and cannot be completely suppressed (Dimberg and Lundqvist, 1990; Dimberg et al., 2002); they even occur in response to subliminally presented emotional expressions (Dimberg et al., 2000). However, there is up to now no experimental empirical evidence answering the question about the neuronal structures involved in the occurrence of such automatic, spontaneous facial mimicry reactions. The present study is a first approach to fill this lack of research by simultaneously acquiring facial electromyography (EMG) and functional magnetic resonance imaging (fMRI).

According to current literature, the neuronal base of (facial) mimicry is presumably the “mirror neuron system” (MNS) (Blakemore and Frith, 2005; Iacoboni and Dapretto, 2006; Niedenthal, 2007). The discovery of mirror neurons dates from studies in the macaque where Giacomo Rizzolatti and colleagues came across a system of cortical neurons in area F5 (premotor cortex in the macaque) and PF [part of the inferior parietal lobule (IPL)] that responded not only when the monkey performed an action, but also when the monkey watched the experimenter performing the same action (di Pellegrino et al., 1992; Gallese et al., 2002). They named their system of neurons the MNS because it appeared that the observed action was reflected or internally simulated within the monkey's own motor system.

There is now evidence that an equivalent system exists in humans. According to a review by Iacoboni and Dapretto (2006), the human MNS should comprise the ventral premotor cortex (vPMC, i.e., the human homolog of the monkey F5 region), the inferior frontal gyrus (IFG) and the IPL. These regions fit nicely to the macaque's MNS. Further mirror neuron activity has been detected in the superior temporal sulcus (STS) (Iacoboni and Dapretto, 2006) which is seen as the main visual input to the human MNS. However, recent studies reveal a slightly more complex picture of the brain areas that show shared activity during observation and execution of the same behavior. In an fMRI study with unsmoothed single subject data, Gazzola and Keysers (2009) examined shared voxels that show increased BOLD activity both during observing and executing an action and found a wide range of areas containing such shared voxels. Those were classical mirroring regions like the vPMC (BA6/44) and the IPL, but also areas beside the MNS like the dorsal premotor cortex (dPMC), supplementary motor area (SMA), middle cingulate cortex (MCC), somatosensory cortex (BA2/3), superior parietal lobule (SPL), middle temporal gyrus (MTG) and the cerebellum. Additionally, Mukamel et al. (2010) reported mirror activities in further brain regions, namely the hippocampus and the parahippocampal gyrus. Yet, Molenberghs et al. (2012) concluded in their broad review of 125 human MNS studies that consistent activations could be found in the classical regions like the IFG, IPL, SPL, and vPMC. They termed these regions the “core network”. However, they also identified activations in other areas depending on the respective modality of the task and stimuli, e.g., for emotional facial expressions enhanced activity in regions known to be involved in emotional processing like the amygdala, insula, and cingulate gyrus.

There are several studies supporting the assumption that the human MNS is involved in facial mimicry. Accordingly, there is evidence for activation in Brodmann area 44 when participants deliberately imitate other people's facial expressions (Carr et al., 2003). van der Gaag et al. (2007) could further show common activations in the IFG and IPL (both termed “classical” MNS sites) as well as the STS, MTG, insula, amygdala, SMA, and somatosensory cortex (called the “extended” MNS) during both the observation and execution (i.e., conscious imitation) of emotional facial expressions. Further studies could show similar relationships between the conscious imitation of facial expressions and activity of parts of the MNS (Leslie et al., 2004; Dapretto et al., 2006; Lee et al., 2006).

Whereas all these studies examined conscious imitation of facial expressions, other authors are interested in the relationship between the MNS and *unconscious* facial mimicry. In a TMS study, Enticott et al. (2008) could show that accuracy in facial emotion recognition was significantly associated with increased motor-evoked potentials during perception of the respective facial expressions. Because facial mimicry is supposed to be related to emotion recognition (Niedenthal et al., 2001; Oberman et al., 2007) the authors interpret this enhanced activation of the MNS as connected to an internal simulation of the observed expression comparable to facial mimicry. On the other hand, Jabbi and Keysers (2008) interpret similar results in a different fashion. They found a causal connection of a prominent part of the MNS, i.e., the IFG, with a region encompassing the anterior insula and the frontal operculum which is known to be responsible for the experience and sharing of emotions like disgust. The authors conclude that this finding reflects a fast and covert motor simulation of perceived facial expressions by the MNS and that this covert simulation might be sufficient to trigger emotional sharing without the need for overt facial mimicry.

These results, however, provide only *indirect* evidence for or against a relation between the MNS and unconscious mimicry. So far, there is only one study directly examining the neuronal correlates of unconscious and spontaneous facial reactions to facial expressions. Studies examining conscious mimicry usually instruct their participants to imitate a seen facial expression deliberately and compare reactions in that condition with those from a passive viewing condition. However, in such a passive viewing condition participants should also show mimicry, i.e. unconscious facial mimicry. Hence, Schilbach et al. (2008) assessed spontaneous facial muscular reactions via EMG and blood oxygen level dependent (BOLD) responses to dynamic facial expressions of virtual characters via fMRI in two separate experiments. Participants in both of their experiments were instructed to just passively view the presented expressions. They found enhanced activity of the precentral cortex, precuneus, hippocampus, and cingulate gyrus in the time window in which non-conscious facial mimicry occurred. Unfortunately, Schilbach et al. (2008) did not assess muscular activity and BOLD response in the same participants and at the same point in time. Thus, there is up to now no certain empirical evidence about the neuronal structures involved in automatic, spontaneous mimicry.

Therefore, the present study is a first approach to investigate whether the MNS is indeed responsible for differences in *unconscious* and *spontaneous* facial mimicry reactions. Following the studies by Gazzola and Keysers (2009), Molenberghs et al. (2012), Mukamel et al. (2010), Schilbach et al. (2008), and van der Gaag et al. (2007) we constructed a single MNS-region of interest (ROI) for the current experiment consisting of following parts of the MNS: IFG, vPMC, IPL, SMA, cingulate cortex, SPL, MTG, cerebellum, somatosensory cortex, STS, hippocampus, parahippocampal gyrus, precentral gyrus, precuneus, insula, amygdala, caudate, and putamen. Activity in this region will be related to participants' congruent facial muscular reactions to examine which parts of the MNS show significant co-activations with the respective facial mimicry.

This question shall be answered via the *simultaneous* measurement of facial muscular activity via (EMG) and the BOLD response via fMRI. To our knowledge, until now no study with such a design has been published. In a first approach, Heller et al. (2011) measured *M. corrugator supercilii* activity in response to affective pictures between interleaved scan acquisitions; that means that they analyzed muscle activity only for time periods in which no echoplanar imaging (EPI) sequences were collected because EPI collection produces intense electromagnetic noise. However, with this method it is only possible to measure the neuronal activity before and after the EMG recordings but not in exactly the same time window in which the facial reactions occur. Furthermore, with such a sequential recording BOLD and EMG are measured in two different contexts. Especially the noise that differs between EPI and non-EPI sequences but also other influences like repeated presentations or the quality of the preceding stimulus are significant differences between the BOLD and the EMG recording phases that hamper a valid detection of connections between brain activations and muscular reactions. Therefore, in the present study we will measure muscular activity and BOLD simultaneously, i.e., during the collection of EPI images.

## Methods

### Participants

Twenty-three right-handed female participants were investigated. Only female subjects were tested because women show more pronounced, but not qualitatively different mimicry effects than male subjects (Dimberg and Lundqvist, 1990). Informed consent was obtained from all subjects prior to participation and is archived by the authors. All participants received 12€ allowance. Three participants had to be excluded from the analysis due to incomplete recordings or insufficient quality of the MRI data. Therefore, analyses were performed for 20 participants, aged between 20 and 30 years (*M* = 23.50, *SD* = 3.05). The experimental protocol was approved by the institution's ethics committee and conforms to the Declaration of Helsinki.

### Stimuli and apparatus

#### Facial stimuli

As facial stimuli avatar facial emotional expressions are used. Avatars (i.e., virtual persons or graphic substitutes for real persons) provide a useful tool for research in emotion and social interactions (Blascovich et al., 2002), because they allow better control over the facial expression and its dynamics, e.g., its intensity and temporal course, than pictures of humans (Krumhuber and Kappas, 2005). Furthermore, due to the possibility to use the same prototypical faces for all types of characters there is no need to control for differences in liking and attractiveness between the conditions and a reduced amount of error variance can be assumed. How successfully avatars can be used as a research tool for studying interactions has been demonstrated by Bailenson and Yee (2005). Subjects rated a digital chameleon, i.e., an avatar which mimics behavior, more favorably even though they were not aware of the mimicry. Thus, an avatar's mimicry created liking comparable to real individuals (Chartrand and Bargh, 1999).

Stimuli were created with *Poser* software (*Curious Labs*, Santa Cruz, CA) and the software extension offered by Spencer-Smith et al. (2001) to manipulate action units separately according to the facial action coding system (Ekman and Friesen, 1978). Notably, Spencer-Smith et al. (2001) could show that ratings of quality and intensity of the avatar emotional expressions were comparable to those of human expressions from the *Pictures of Facial Affect* (Ekman and Friesen, 1976).

**Figure 1:**
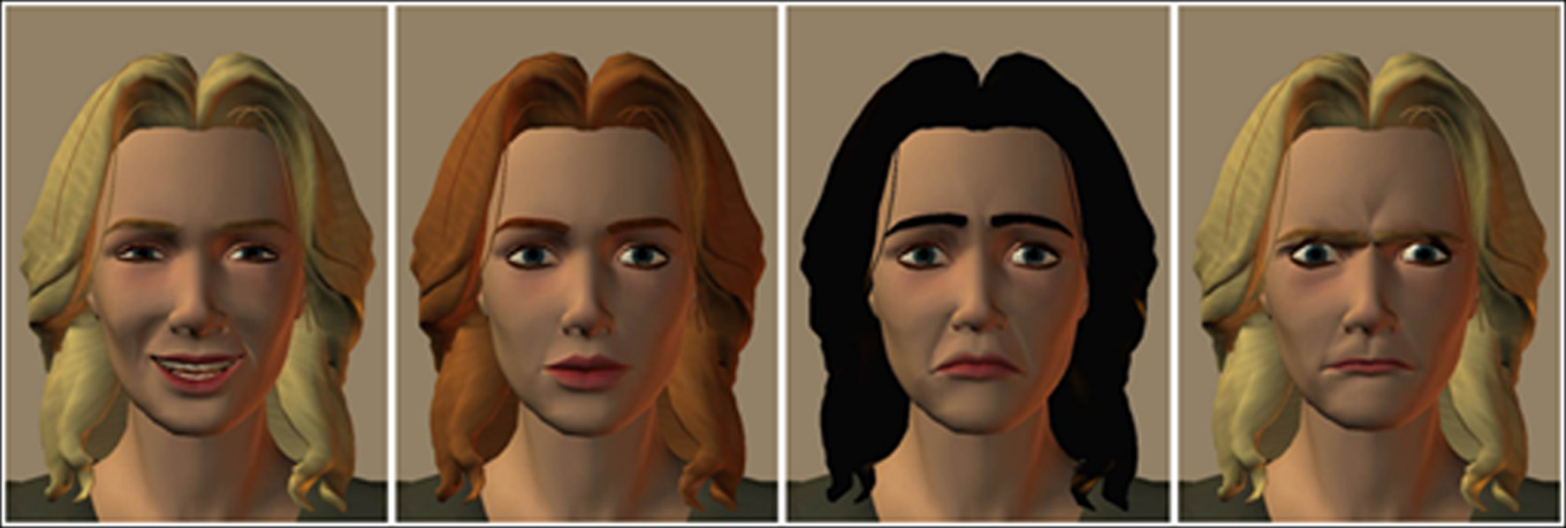
**Examples of avatars with different emotional facial expressions (happy, neutral, sad, angry)**.

The stimuli were presented on a light gray background via MRI-compatible goggles (VisuaStim; Magnetic Resonance Technologies, Northridge, CA). Four facial expressions were created from a prototypic female and a prototypic male face: a neutral, a happy, a sad and an angry expression (for details see Spencer-Smith et al., 2001). Each male and female emotional expression was then combined with three types of hairstyles (blond, brown, and black hair), resulting in twenty-four stimuli (for examples see Figure 1).

#### Facial EMG

**Figure 2:**
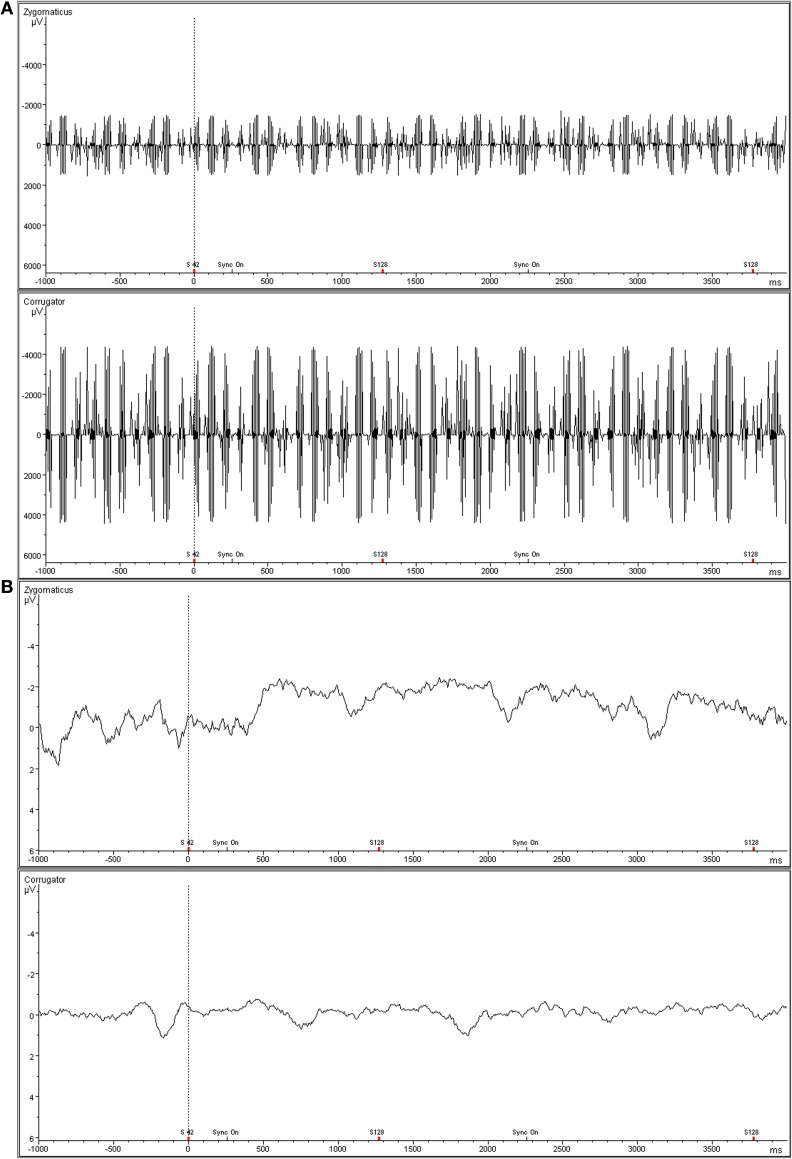
**Representative snapshot of raw zygomaticus and corrugator EMG data acquired simultaneously with fMRI. (A)** Top panel is raw, unfiltered EMG data. **(B)** Bottom panel shows filtered EMG data.

Activity of the *M. zygomaticus major* (the muscle involved in smiling) and the *M. corrugator supercilii* (the muscle responsible for frowning) was recorded on the left side of the face using bipolar placements of MRI-compatible electrodes (MES Medizinelektronik GmbH, Munich, Germany) according to the guidelines established by Fridlund and Cacioppo (1986). In order to cover the recording of muscular activity participants were told that skin conductance would be recorded (see e.g., Dimberg et al., 2000). The EMG raw signal was measured with an MRI-compatible BrainAmp ExG MR amplifier (Brain Products Inc., Gilching, Germany), digitalized by a 16-bit analogue-to-digital converter, and stored on a personal computer with a sampling frequency of 5000 Hz. The EMG data were post-processed offline using Vision Analyzer software (Version 2.01, Brain Products Inc., Gilching, Germany). EMG data recorded in the MR scanner is contaminated with scan-pulse artifacts, originating from the switching of the radio-frequency gradients. To remove these artifacts the software applies a modified version of the average artifact subtraction method (AAS) described by Allen et al. (2000). This MRI-artifact correction has originally been developed for combined EEG/fMRI recordings (for applications see e.g., Jann et al., 2008; Musso et al., 2010) and can now also be applied for EMG data. Thereby, a gradient artifact template is subtracted from the EMG using a baseline corrected average of all MR-intervals. Data were then down-sampled to 1000 Hz. Following gradient artifact correction raw data were rectified and filtered with a 30 Hz low cutoff filter, a 500 Hz high cutoff filter, a 50 Hz notch filter, and a 125 ms moving average filter. The EMG scores are expressed as change in activity from the pre-stimulus level, defined as the mean activity during the last second before stimulus onset. Trials with an EMG activity above 8 μV during the baseline period and above 30 μV during the stimuli presentation were excluded (less than 5%). Before statistical analysis, EMG data were collapsed over the 12 trials with the same emotional expression, and reactions were averaged over the 4 s of stimulus exposure. An example snapshot of the raw and the filtered zygomaticus and corrugator EMG data can be seen in Figure 2.

### Image acquisition

Image acquisition followed the standard procedure in our lab (Gerdes et al., 2010; Mühlberger et al., 2011): Functional and structural MRI was performed with a Siemens 1.5 T MRI whole body scanner (SIEMENS Avanto) using a standard 12-channel head coil and an integrated head holder to reduce head movement. Functional images were obtained using a T2^*^—weighted single-shot gradient EPI sequence (TR: 2500 ms, TE: 30 ms, 90° flip angle, FOV: 200 mm, matrix: 64 × 64, voxel size: 3.1 × 3.1 × 5 mm^3^). Each EPI volume contained 25 axial slices (thickness 5 mm, 1 mm gap), acquired in interleaved order, covering the whole brain. The orientation of the axial slices was parallel to the AC–PC line. Each session contained 475 functional images. The first eight volumes of each session were discarded to allow for T1 equilibration. In addition, a high-resolution T1-weighted magnetization-prepared rapid gradient-echo imaging (MP-RAGE) 3D MRI sequence was obtained from each subject (TR: 2250 ms, TE: 3.93 ms, 8° flip angle, FOV: 256 mm, matrix: 256 × 256, voxel size: 1 × 1 × 1 mm^3^).

### Image preprocessing and analyses

Data were analyzed by using Statistical Parametric Mapping software (SPM8; Wellcome Department of Imaging Neuroscience, London, UK) implemented in Matlab R2010a (Mathworks Inc., Sherborn, MA, USA). Functional images were slice-time corrected and realignment (b-spline interpolation) was performed (Ashburner and Friston, 2003). To allow localization of functional activation on the subjects' structural MRIs, T1-scans were coregistered to each subject's mean image of the realigned functional images. Coregistered T1 images were then segmented (Ashburner and Friston, 2005) and in the next step, EPI images were spatially normalized into the standard Montreal Neurological Institute (MNI) space using the normalization parameters obtained from the segmentation procedure (voxel size 2 × 2 × 2 mm^3^) and spatially smoothed with an 8 mm full-width-half-maximum (FWHM) Gaussian kernel. Each experimental condition (happy, neutral, sad, and angry) and the fixation periods were modeled by a delta function at stimulus onset convolved with a canonical hemodynamic response function. Parameter estimates were subsequently calculated for each voxel using weighted least squares to provide maximum likelihood estimates based on the non-sphericity assumption of the data in order to get identical and independently distributed error terms. Realignment parameters for each session were included to account for residual movement related variance. Parameter estimation was corrected for temporal autocorrelations using a first-order autoregressive model.

For each subject, the following *t*-contrasts were computed: “happy > fixation cross”, “sad > fixation cross”, “angry > fixation cross”, “happy + sad + angry > fixation cross”, “happy > neutral”, “sad > neutral” and “angry > neutral”. We did not analyze the contrast “neutral > fixation cross” because no facial mimicry reactions are expected in response to neutral faces and thus no neural correlates of facial mimicry can be computed. For a random effect analysis, the individual contrast images (first-level) were used in a second-level analysis. FMRI data were analyzed specifically for the ROI (MNS-ROI, see above). To investigate the brain activity in relation to the facial muscular reactions, we performed six regression analyses with estimated BOLD responses of individual first-level contrast images (“happy > fixation cross”, “happy > neutral”, “sad > fixation cross”, “sad > neutral”, “angry > fixation cross”, “angry > neutral”) as dependent variable and the according congruent facial reactions (zygomaticus to happy expressions, corrugator to sad expressions, corrugator to angry expressions) as predictors.

The WFU Pickatlas software (Version 2.4, Wake Forest University, School of Medicine, NC) was used to conduct the small volume correction with pre-defined masks in MNI-space (Tzourio-Mazoyer et al., 2002; Maldjian et al., 2003, 2004). For the ROI analysis, alpha was set to *p* = 0.05 on voxel-level, corrected for multiple comparisons (family-wise error–FWE) and meaningful clusters exceeding 5 significant voxels.

### Procedure

After arriving at the laboratory, participants were informed about the procedure of the experiment and were asked to give informed consent. They were told that the experiment was designed to study the avatars' suitability for a future computer game to cover the true purpose of the experiment in order to avoid deliberate manipulation of the facial reactions. The EMG electrodes were then attached and participants were placed in the MRI scanner. Following this the functional MRI session started. Each of the four expressions was repeated 24 times, i.e., a total of 96 facial stimuli were presented in a randomized order. Faces were displayed for 4000 ms after a fixation-cross had been presented for 2000 ms to ensure that participants were focusing on the center of the screen. The inter-trial interval varied randomly between 8750 and 11,250 ms. Participants were instructed to simply view the pictures without any further task. After the functional MRI the structural MRI (MP-RAGE) was recorded. Then, participants were taken out of the scanner and electrodes were detached. Finally participants completed a questionnaire regarding demographic data, were debriefed, paid and thanked.

## Results

### EMG measures

A repeated measures analysis of variance with the within-subject factors muscle (*M. zygomaticus major* vs. *M. corrugator supercilii*) and emotion (happy vs. neutral vs. sad vs. angry) was conducted. A main effect of emotion, *F*_(3, 17)_ = 4.17, *p* = 0.02, η_*p*_^2^ = 0.20, and a significant Muscle × Emotion effect, *F*_(3, 17)_ = 9.38, *p* < 0.01, η_*p*_^2^ = 0.33, occurred. The main effect muscle did not gain significance, *p* > 0.36. To further specify the Muscle × Emotion interaction, separate follow up ANOVAs for the *M. zygomaticus major* and the *M. corrugator supercilii* were calculated.

#### M. zygomaticus major

**Figure 3:**
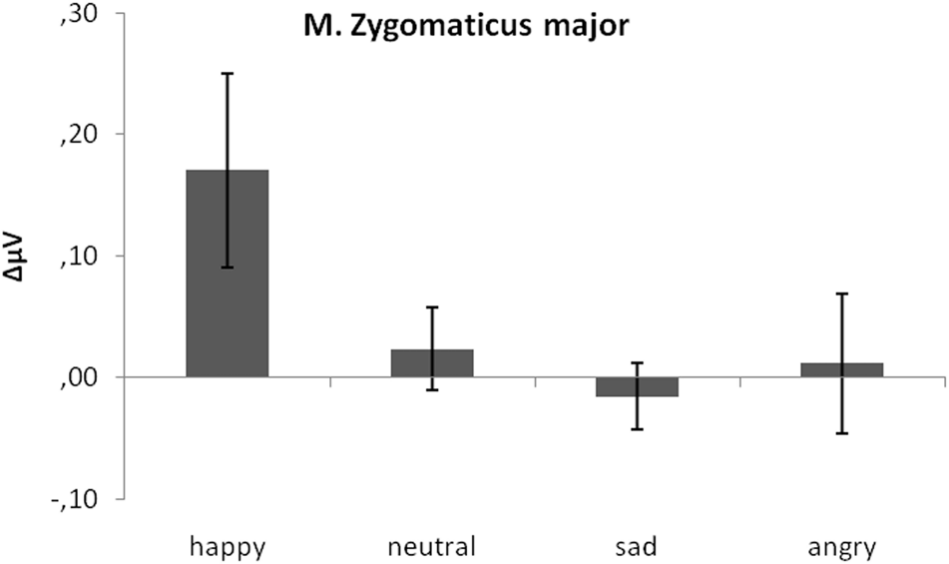
**Mean EMG change from baseline in μV for *M. zygomaticus major* in response to happy, neutral and sad faces.** Error bars indicate standard errors of the means.

As predicted, activity in *M. zygomaticus major* was larger to happy compared to neutral, sad, and angry faces (see Figure 3). This was verified by a significant emotion effect, [*F*_(3, 17)_ = 3.91, *p* = 0.04, η_*p*_^2^ = 0.176]. Following *t*-tests revealed a significant difference between *M. zygomaticus major* reactions to happy faces (*M* = 0.17) as compared to neutral (*M* = 0.02), *t*(19) = 2.64, *p* = 0.02, sad (*M* = −0.02), *t*(19) = 2.09, *p* = 0.05, and angry expressions (*M* = 0.01), *t*(19) = 3.57, *p* < 0.01. No other significant differences were observed, all *p*s > 0.41.

#### M. corrugator supercilii

**Figure 4:**
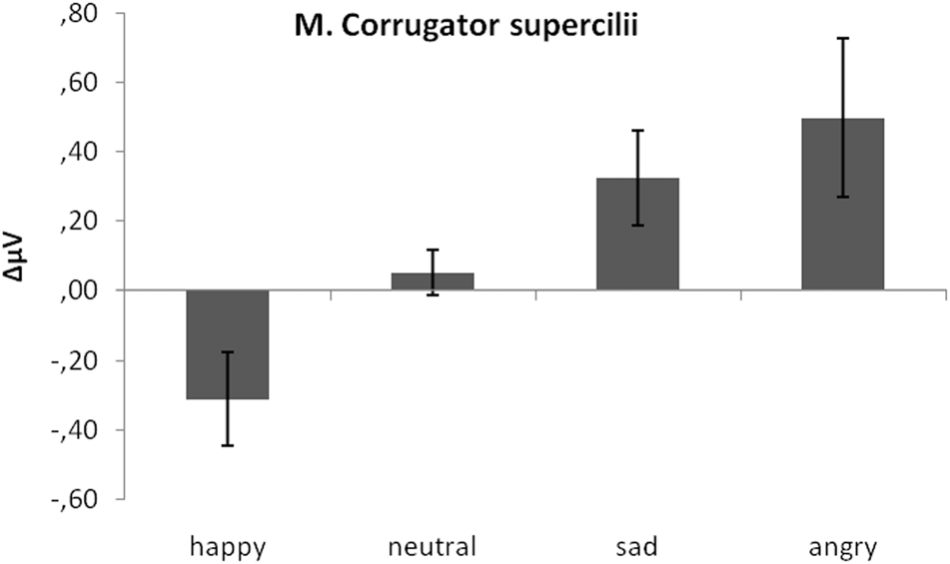
**Mean EMG change from baseline in μV for *M. corrugator supercilii* in response to happy, neutral, and sad faces.** Error bars indicate standard errors of the means.

As predicted, activity in *M. corrugator supercilii* was larger to sad and angry faces as compared to neutral and positive faces (see Figure 4). This was verified by a significant emotion effect, [*F*_(3, 17)_ = 7.58, *p* < 0.01, η_*p*_^2^ = 0.28]. Following *t*-tests revealed a significant difference between *M. corrugator supercilii* reactions to sad faces (*M* = 0.32) as compared to happy (*M* = −0.31), *t*(19) = 3.12, *p* < 0.01, and neutral expressions (*M* = 0.05), *t*(19) = 2.56, *p* = 0.02. In a similar vein, reactions to angry faces (*M* = 0.50) differed from reactions to happy, *t*(19) = 2.91, *p* < 0.01, and neutral faces, *t*(19) = 2.41, *p* = 0.03. Furthermore, *M. corrugator supercilii* reactions in response to happy expressions differed from reactions to neutral faces, *t*(19) = 2.53, *p* = 0.02. Reactions to sad and angry faces did not differ, *p* > 0.13.

Additionally, one-sample *t*-tests against zero revealed that the *M. zygomaticus major* reaction to happy faces was indeed an increase in activity, *t*(19) = 2.13, *p* = 0.04. Furthermore, the *M. corrugator supercilii* reaction to happy expressions was a significant decrease in activity, *t*(19) = 2.33, *p* = 0.03, whereas reactions to sad and angry faces both occurred to be significant activity increases, *t*(19) = 2.35, *p* = 0.03 and *t*(19) = 2.19, *p* = 0.04. Therefore, all these reactions can be seen as congruent facial reactions. All other reactions did not differ from zero, all *p*s > 0.41.

### fMRI data

ROI analyses were performed for the contrasts comparing the brain activation during viewing of emotional expressions with the activation during the fixation crosses, i.e., “expression” > “fixation cross”. These analyses revealed for all expression contrasts (“happy > fixation cross”, “sad > fixation cross”, “angry > fixation cross”) significant activations (FWE-corrected, *p* < 0.05, minimum cluster size of *k* = 5 voxels) in numerous classical (core) as well as extended parts of the MNS. Those were IFG, IPL, MTG, STS, precentral gyrus, cerebellum, hippocampus, amygdala, caudate, putamen, insula, and posterior cingulate cortex (PCC). Additionally, the contrast “happy > fixation cross” revealed activations in the MCC, the parahippocampal gyrus, the precuneus and the SMA. The contrast “sad > fixation cross” revealed further significant activations in the precuneus. ROI analyses for the contrast “happy + sad + angry > fixation cross” as well as all contrasts comparing the emotional expressions with activation during the neutral expression (“happy > neutral”, “sad > neutral” and “angry > neutral”) did not reveal any significant clusters (FWE-corrected, *p* < 0.05, minimum cluster size of *k* = 5 voxels).

#### Regression analyses

**Figure 5:**
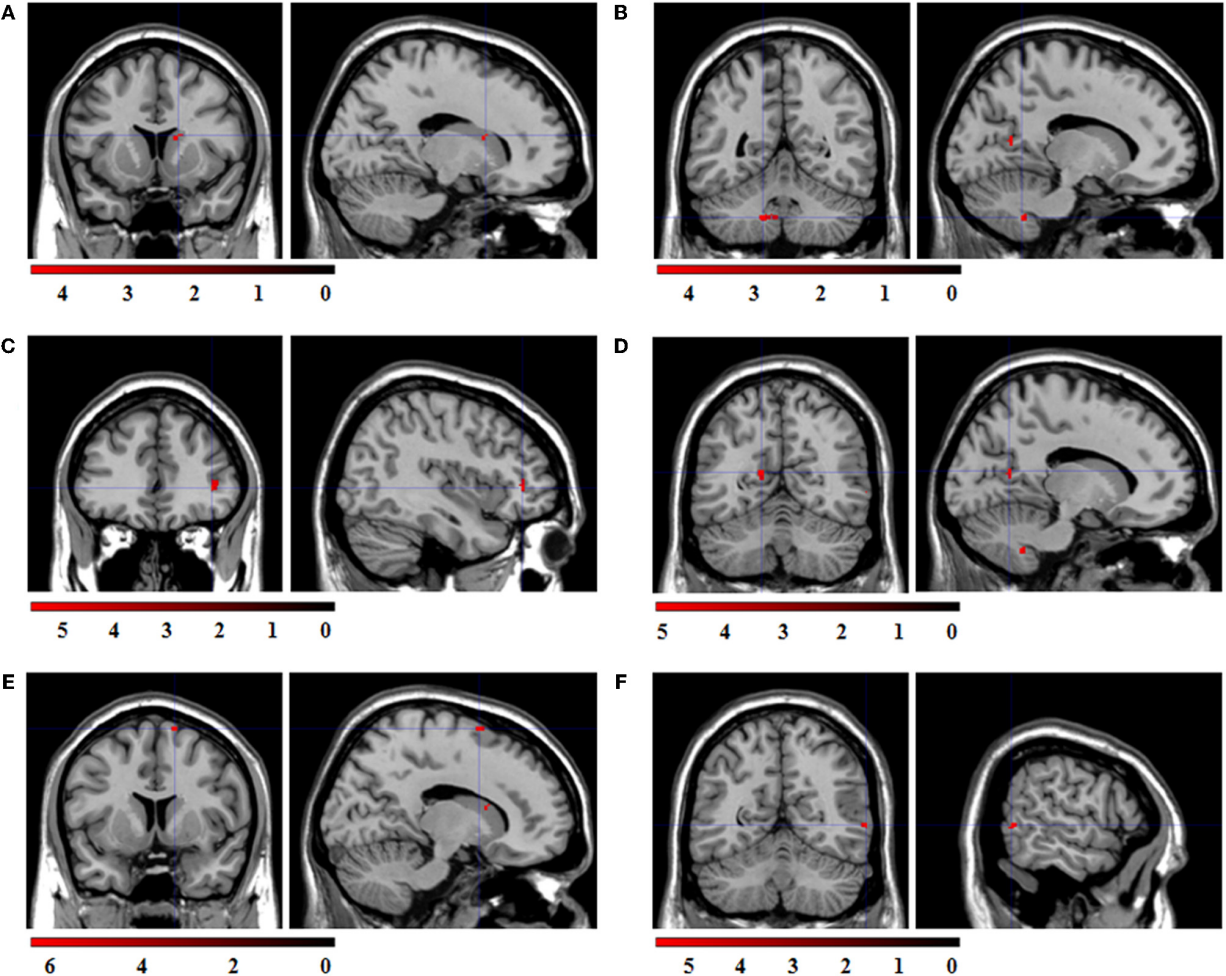
**Statistical parametric maps for the ROI regression analyses with BOLD-contrast “happy > fixation cross” as dependent variable and zygomaticus reactions to happy expressions as predictor.** FWE-corrected, alpha = 0.05, *k* ≥ 5 voxels. Coordinates *x, y*, and *z* are given in MNI space. Color bars represent the *T*-values. **(A)** Significant co-activation in the right caudate, (*x* = 16, *y* = 12, *z* = 16; *t* = 4.55; *k* = 6 voxel). **(B)** Significant co-activation in the left cerebellum, (*x* = −14, *y* = −52, *z* = −42; *t* = 4.58; *k* = 29 voxel). **(C)** Significant co-activation in the right inferior frontal gyrus, (*x* = 40, *y* = 38, *z* = 2; *t* = 5.82; *k* = 18 voxel). **(D)** Significant co-activation in the left posterior cingulate cortex, (*x* = −14, *y* = −60, *z* = 14; *t* = 5.03; *k* = 6 voxel). **(E)** Significant co-activation in the right supplementary motor area, (*x* = 14, *y* = 8, *z* = 70; *t* = 6.27; *k* = 6 voxel). **(F)** Significant co-activation in the right middle temporal cortex, (*x* = 60, *y* = −58, *z* = 2; *t* = 5.44; *k* = 5 voxel).

Regression analyses with the contrasts “expression > fixation cross” as dependent and the respective congruent facial reactions, measured simultaneously via EMG, as predictor variable were computed to investigate which brain activations were related to the occurrence of facial mimicry. The corresponding ROI regression analysis with BOLD contrast “happy > fixation cross” as dependent variable and zygomaticus reactions to happy expressions as predictor revealed significant co-activations in the caudate, cerebellum, IFG, PCC, SMA, and MTG (see Figure 5).

**Figure 6:**
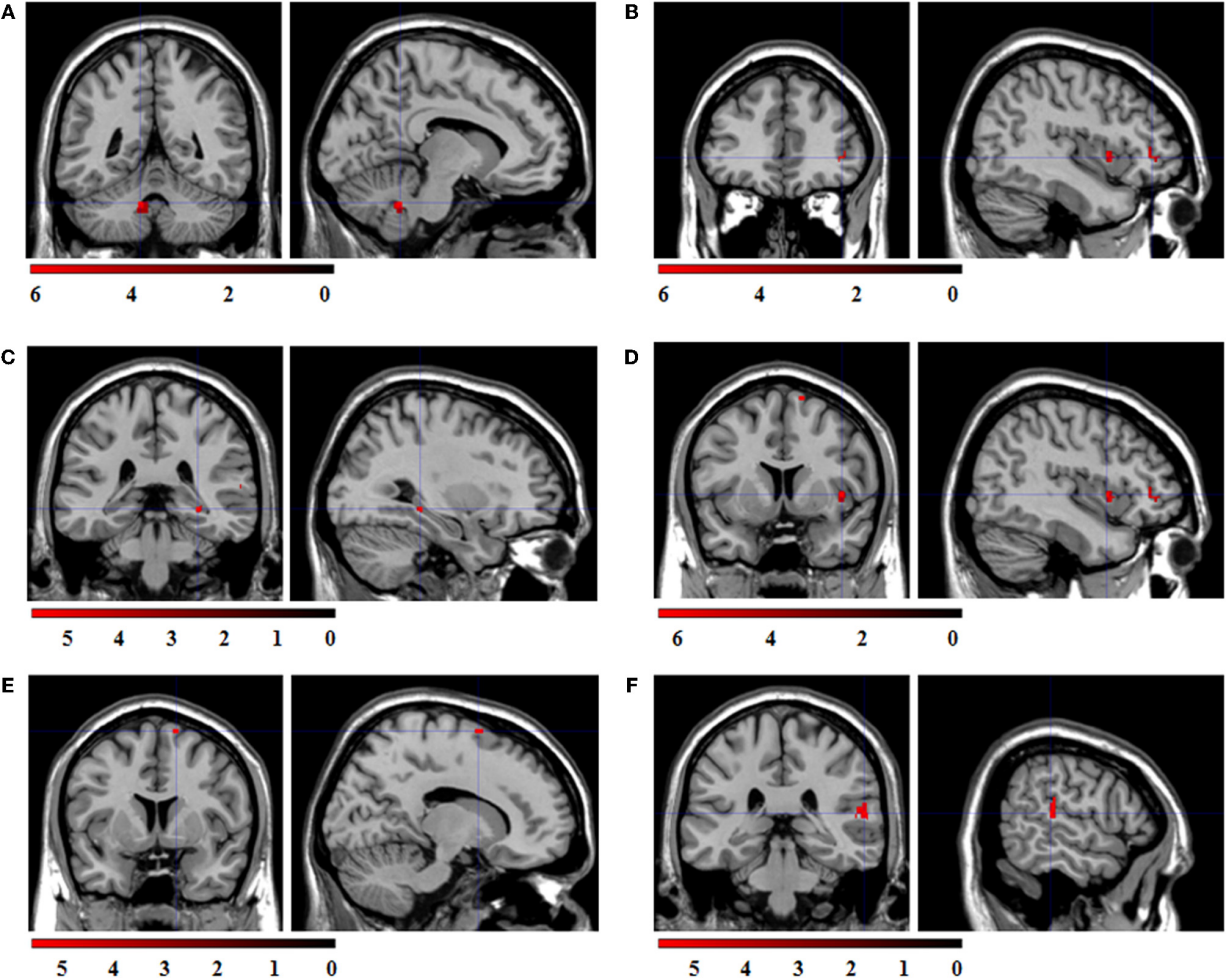
**Statistical parametric maps for the ROI regression analyses with BOLD-contrast “angry > fixation cross” as dependent variable and corrugator reactions to angry expressions as predictor.** FWE-corrected, alpha = 0.05, *k* ≥ 5 voxels. Coordinates *x, y*, and *z* are given in MNI space. Color bars represent the *T*-values. **(A)** Significant co-activation in the left cerebellum, (*x* = −10, *y* = −48, *z* = −32; *t* = 6.24; *k* = 43 voxel). **(B)** Significant co-activation in the right inferior frontal gyrus, (*x* = 42, *y* = 40, *z* = 0; *t* = 6.25; *k* = 25 voxel). **(C)** Significant co-activation in the right hippocampus, (*x* = 30, *y* = −34, *z* = −6; *t* = 5.91; *k* = 8 voxel). **(D)** Significant co-activation in the right insula, (*x* = 42, *y* = 8, *z* = 2; *t* = 6.54; *k* = 19 voxel). **(E)** Significant co-activation in the right supplementary motor area, (*x* = 14, *y* = 6, *z* = 70; *t* = 5.26; *k* = 5 voxel). **(F)** Significant co-activation in the right superior temporal sulcus, (*x* = 58, *y* = −32, *z* = 12; *t* = 5.65; *k* = 35 voxel).

ROI regression analysis with BOLD contrast “sad > fixation cross” as dependent and corrugator reactions to sad expressions as predictor variable revealed no significant co-activations. ROI regression analysis with the BOLD contrast “angry > fixation cross” as dependent variable and the corrugator reactions to angry expressions as predictor variable revealed significant co-activations in the cerebellum, IFG, hippocampus, insula, SMA, and STS (see Figure 6).

Finally, the three ROI regression analyses with BOLD contrasts “emotional expression > neutral expression” (“happy > neutral”, “sad > neutral”, “angry > neutral”) as dependent and the according congruent facial reactions as predictors revealed no significant co-activations.

## Discussion

The present experiment is a first approach revealing the neuronal structures responsible for differences in automatic and spontaneous facial mimicry reactions in a clear and experimental fashion. In a first step it was shown that a broad network of regions with mirroring properties is active during the perception of emotional facial expressions. This network included for all expressions the IFG, IPL, MTG, STS, precentral gyrus, cerebellum, hippocampus, amygdala, caudate, putamen, insula, and PCC as well as for happy expressions the MCC, the parahippocampal gyrus, the precuneus and the SMA, and for sad expressions additionally the precuneus. These findings replicate earlier studies showing an involvement of both classical and “extended” mirror neuron regions in the observation and execution of (facial) movements (e.g., van der Gaag et al., 2007; Molenberghs et al., 2012).

More importantly, in a second step we explored which of these brain regions show a direct relation with the individual strength of facial mimicry reactions by regressing the BOLD data on the simultaneously measured facial EMG reactions. The EMG measurement proved to deliver reliable and significant data comparable to earlier studies on attitude effects on facial mimicry (Likowski et al., 2008). It was found that both zygomaticus reactions to happy expressions and corrugator reactions to angry faces correlate significantly with activations in the right IFG, right SMA, and left cerebellum. Stronger zygomaticus reactions to happy faces were further associated with an increase in activity in the right caudate, the right MTG as well as the left PCC. Corrugator reactions to angry expressions were also correlated with the right hippocampus, the right insula, and the right STS. This shows that although a wide range of regions assumed to belong to the core and the extended MNS is active during the observation of emotional facial expressions only a small number actually seems to be related to the observed strength of facial mimicry. The correlated regions are on the one hand regions concerned with the perception and execution of facial movements and their action representations. For example, the STS codes the visual perception, the MTG is responsible for the sensory representation (Gazzola and Keysers, 2009), the IFG is responsible for coding the goal of the action (Gallese et al., 1996), and the SMA is concerned with the execution of the movement (Cunnington et al., 2005). On the other hand, we also observed associations of mimicry and regions involved in emotional processing. We found co-activations in the insula which connects the regions for action representation with the limbic system (Carr et al., 2003) and the caudate and the cingulate cortex which are involved in processing positive and negative emotional content (Mobbs et al., 2003; Vogt, 2005).

These results fit nicely with assumptions of the MNS. It is widely assumed that the function of the MNS is to decode and to understand other people's actions (Carr et al., 2003; Rizzolatti and Craighero, 2004; Iacoboni and Dapretto, 2006; but see Decety, 2010; Hickok and Hauser, 2010 for a discussion). Accordingly, Carr et al. (2003) suggest that the activation of areas concerned with action representation and emotional content helps to resonate, simulate and thereby recognize the emotional expression and to empathize with the sender. This assumption overlaps with theories on the purpose of facial mimicry. According to embodiment theories congruent facial reactions are part of the reenactment of the experience of another person's state (Niedenthal, 2007). Specifically, embodiment theories assume that during an initial emotional experience all the sensory, affective and motor neural systems are activated together. This experience leads to interconnections between the involved groups of neurons. Later on, when one is just thinking about the event or perceiving a related emotional stimulus, the activated neurons in one system spread their activity through the interconnections that were active during the original experience to all the other systems. Thereby the whole original state or at least the most salient parts of the network can be reactivated (Niedenthal, 2007; Oberman et al., 2007; Niedenthal et al., 2009). Embodiment theories state that looking at an emotional facial expression means reliving past experience associated with that kind of face. Thus, perceiving an angry face can lead to tension in the muscles used to strike, a rise in blood pressure or the enervation of facial muscles involved in frowning (Niedenthal, 2007). Accordingly, congruent facial reactions reflect an internal simulation of the perceived emotional expression. The suggested purpose of such simulation is like for mirror neurons understanding the actor's emotion (Wallbott, 1991; Niedenthal et al., 2001; Atkinson and Adolphs, 2005).

Contrary to expectations, no correlations of MNS activities and facial mimicry were found in response to sad expressions. The reason for that is unclear. We observed proper mimicry reactions in the corrugator muscle, comparable to those to angry expressions. Also the number of significant clusters and their respective sizes were comparable for all emotional expressions. Maybe the low arousal of sad facial expressions (see e.g., Russell and Bullock, 1985) compared to other negative stimuli hampered the detection of co-activations in this case. However, this is pure speculation and should be investigated in further studies.

The contrasts “emotional expression > neutral expression” as well as the regression analyses with these contrasts revealed no significant clusters in the reported ROIs. We attribute this to the finding that many of the regions involved in processing the emotional expressions (happy, sad, angry) are also activated during perception of the neutral expressions (as revealed by the contrast “neutral > fixation”). Such overlapping clusters probably reflect activations of general face processing and might be responsible for the lower contrast effects and thereby also for lower variances which presumably prevented our regressions from showing valid and significant effects. One might now argue that the overlap in activations in response to emotional as well as neutral expressions suggests that we just observed general and unspecific face processing regions. Importantly, we can proof that this is not the case. The fact that our regression results are only significant for the congruent pairings of BOLD and muscular activation but not for incongruent pairings (like e.g., BOLD to happy expressions and corrugator activity to sad expressions) clearly shows that we observed specific relations of regions with mirror properties and facial muscular reactions. Furthermore, we can conclude from the non-significant contrast “happy + sad + angry > fixation cross” that the effects of the three separate contrasts “happy > fixation cross”, “sad > fixation cross” and “angry > fixation cross” appear to be rather specific regarding the locations of the relevant clusters.

Taken together, the results of this experiment are the first to show successful simultaneous recording of facial EMG and functional MRI. Thus, it was possible to examine which specific parts of the MNS were associated with differences in the occurrence of facial mimicry, i.e., the strength of congruent facial muscular reactions in response to emotional facial expressions. It was found that mimicry reactions correlated significantly with prominent parts of the classic MNS as well as with areas responsible for emotional processing. These results and the here introduced methods for simultaneous measurement may provide a promising starting point for further investigations on moderators and mediators of facial mimicry.

### Conflict of interest statement

The authors declare that the research was conducted in the absence of any commercial or financial relationships that could be construed as a potential conflict of interest.
